# Cataract: optimising and monitoring surgical outcomes

**Published:** 2025-12-02

**Authors:** Lila Raj Puri

**Affiliations:** 1 Medical Advisor, Asia: The Fred Hollows Foundation, London, UK.

**Improving surgical technique, monitoring, and pre- and postoperative examination ensures a future where cataract surgery is not just widely available, but also effective, equitable, and trusted.**

**Figure:**
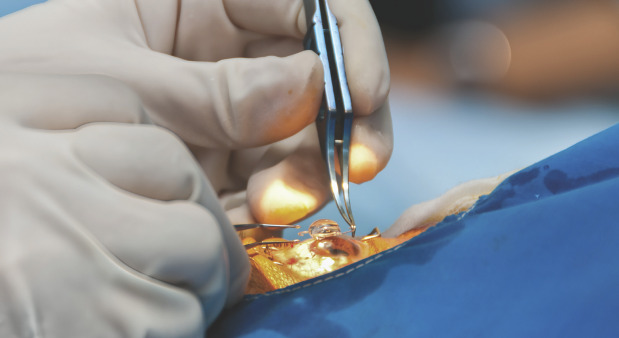
Preparing to insert an intraocular lens during cataract surgery. INDONESIA

© I WAYAN HELMY GIANA, THE JOHN FAWCETT FOUNDATION, COURTESY OF IAPB CC BY-NC-SA 4.0

Cataract remains the leading cause of blindness worldwide, disproportionately affecting people in low- and/or middle-income countries. In recent decades, significant progress has been made in scaling up cataract surgical services, leading to remarkable increases in surgical volumes. However, surgical numbers alone do not equate to improved visual outcomes or quality of life. As discussed in our previous issue (volume 38, issue 127) poor refractive outcomes after surgery undermine community trust, discourage service uptake, and waste already limited resources.

For patients, the difference between ‘sight restored’ and ‘sight compromised’ can be the difference between independence and disability. With today’s techniques, including manual small incision cataract surgery (MSICS) and phacoemulsification, the goal must be to optimise outcomes, ensuring that every patient, regardless of setting, receives safe, high-quality care.

## The changing landscape of cataract surgery

Modern cataract surgery has moved away from large-incision extracapsular extraction, which required sutures, to sutureless small-incision and phacoemulsification techniques. Phacoemulsification, with its precision, rapid recovery, and excellent refractive predictability, has become the standard of care in high-income and urban settings.^1^ However, the benefits of phacoemulsification come with prerequisites: costly equipment and the need for reliable electricity, high-quality consumables, and extensive training. In resource-limited environments, these requirements can make phacoemulsification impractical or unsustainable at scale.

In contrast, MSICS provides a significantly more cost-effective alternative. When performed with skill and precision, MSICS achieves outcomes comparable to phacoemulsification, even in advanced or brunescent cataracts, where phacoemulsification may pose additional risks.^1^,^2^ Its adaptability, speed, and resilience to infrastructural limitations make MSICS the most appropriate technique for large-scale blindness prevention in lower-resource settings.

In this issue, we focus on the following key components of cataract surgical quality: preoperative assessment, surgical performance – with a focus on MSICS surgery – monitoring and auditing, and postoperative examination. Only by embedding quality at every stage can eye health programmes ensure long-term trust in cataract services and achieve the vision of effective Cataract Surgical Coverage (eCSC) – thereby contributing to the elimination of avoidable blindness.

## Preoperative assessment

The risk of surgical complications can be minimised if cataract teams carry out a thorough preoperative assessment and assign a surgeon with the appropriate level of experience. Such preparation reduces intraoperative surprises, improves patient understanding, and ensures smoother postoperative recovery.^1^ Yet, in many lower-resource settings, this step is either rushed or inadequately performed due to patient load or resource constraints.

Coexisting ocular conditions, such as corneal scarring, glaucoma, uveitis, or retinal pathology, should be identified preoperatively, as these may affect prognosis. Patients should also receive counselling about postoperative expectations, medications, and follow-up.

## Quality considerations during surgery

The surgical act is where the greatest opportunity lies to secure a good visual outcome. Good outcomes depend on meticulous preoperative evaluation, patient counselling, accurate biometry for all patients, an effective infection control policy, skilful surgical execution, and good postoperative management and follow-up. Critical quality considerations during surgery should include adherence to aseptic protocols, consistency in surgical technique through standardised surgical protocols, and continued training (especially for junior surgeons), skills development and mentorship, and the use of appropriate technique.

Even with the best systems, complications are inevitable. The measure of a high-quality service is not whether complications occur, but how effectively they are managed. This should include immediate recognition and management, training surgeons to recognise complications such as posterior capsule rupture or vitreous loss, and equipping facilities with the necessary instruments and consumables for safe management. For complications beyond a surgeon’s capacity, timely referral to higher-level centres must be streamlined.

## Postoperative follow-up: closing the loop

Surgery does not end when the last suture is tied or the wound is sealed. Postoperative follow-up is essential for the early detection and management of complications such as corneal oedema, raised intraocular pressure (IOP), or infection. It is also an opportunity to remind patients about returning for follow-up visits and the importance of adhering with medication and guidance. Ultimately, follow-up ensures optimal visual rehabilitation and better patient satisfaction.

Follow-up should be structured: a review on day 1 to check wound integrity and IOP, a visit at around 1 week to monitor healing, and an appointment for refraction at 4–6 weeks. However, many cataract programmes discharge patients after one or two days, with limited follow-up due to travel difficulties or resource constraints. Community-based models such as outreach follow-up, collaboration with primary health workers, or the use of teleophthalmology, can bridge gaps in these settings.

## Surgical outcome monitoring and auditing

The measure of success is not the number of operations performed, but the quality of sight restored.

In lower-resource settings, implementing surgical auditing systems is often resisted due to concerns about workload, fear of judgment, or a lack of infrastructure. However, without objective measurement, quality cannot be improved.

An effective auditing system includes routine data collection for every operation, constructive feedback to the surgical team, benchmarking against international standards, and integration with national programmes to ensure accountability. Cataract surgical outcome monitoring (CSOM) is one example of an auditing system. It involves routine documentation of intraoperative events and visual outcomes. Establishing CSOM systems allows services to identify performance gaps and design targeted training or improvement of processes.

Regular reviews of the data help identify individual or institutional performance gaps. It also facilitates comparison with global benchmarks, such as the World Health Organization’s recommendation that at least 80% of operations achieve presenting visual acuity of 6/12 or better at 4–12 weeks after surgery.^2^

All audits should be conducted in an environment that is focused on learning, not punishment. When they are shared transparently, audits can build a culture of excellence and confidence among surgical teams.

## Conclusion

Cataract surgery in lower resource settings has moved beyond the era of ‘surgery at any cost.’ Communities now expect and deserve good-quality vision after surgery. Community-based programmes should prioritise not just high-volume but also high-quality surgery, ensuring long-term trust in cataract services. By focusing on preoperative evaluation, including basic ocular examination and IOL power calculation; safe, standardised surgical practice with adherence to asepsis; meticulous postoperative management and good follow-up; and continuous audit with constructive feedback, eye care programmes can ensure that every cataract operation delivers lasting visual improvement. This allows cataract programmes in low-resource settings to move towards a future where cataract surgery is not just widely available, but consistently effective, equitable, and trusted.
